# Supplementary material for: What factors shape an individual’s probability to be enrolled in a professionally-managed community-based health insurance? Results from a cross-sectional case-control study in two districts in Mali
Source: PLOS Glob Public Health. 2025 Jul 8;5(7):e0004892. doi: 10.1371/journal.pgph.0004892 (PMC12237063; doi:10.1371/journal.pgph.0004892)
Supplement: S1 Table — The BIC and AIC criteria are mathematical methods for assessing the suitability of a model to the data from which they were generated [53]. The model with smallest values of the BIC or AIC tests is the one that adjusts the data better. The LL test is a measure of the suitability of a model; the higher the value, the better the model. The ICC expresses the proportion of total variance at the common or village level [54]. The greater the value of the ICC, the more the characteristics of the groups (municipalities, villages) are different, and therefore, the model with mixed effects is appropriate. The results of AIC, BIC and LL tests do not discriminate between different models. (DOCX) [file pgph.0004892.s001.docx]

S1 Table : Statistical tests of model comparisons

|  |  | Level 1 |  | Level 2 | | | | |
| --- | --- | --- | --- | --- | --- | --- | --- | --- |
|  |  | Model 1 |  | Model 2 |  | Model 3 |  | Model 4 |
|  |  |  |  | Indiv. |  | Village |  | Com. |
|  |  |  |  |  |  |  |  |  |
| Chi 2 |  | 126,7 |  | 106,68 |  | 106,68 |  | 106,68 |
| BIC |  | 1352,89 |  | 1352,89 |  | 1352,89 |  | 1352,89 |
| AIC |  | 1245,59 |  | 1245,59 |  | 1245,59 |  | 1245,59 |
| LL |  | —600,79 |  | —600,79 |  | —600,79 |  | —600,79 |
| ICC | | — |  | 4,87E-33 |  | 3,23E-36 |  | 2,82E-33 |
|  |  | Level 3 | | | | | Level 4 | |
|  |  | Model 5 |  | Model 6 |  | Model 7 |  | Model 8 |
|  |  | Vil-Indiv. |  | Com. – Indiv. |  | Com. -Vill |  | Com. -Vill-Indiv |
| Chi 2 |  | 106,68 |  | 106,68 |  | 106,68 |  | 106,68 |
| BIC |  | 1352,89 |  | 1352,89 |  | 1352,89 |  | 1352,89 |
| AIC |  | 1245,59 |  | 1245,59 |  | 1245,59 |  | 1245,59 |
| LL |  | —600,79 |  | —600,79 |  | —600,79 |  | —600,79 |
| ICC | Village | 6,07E-35 | Com. | 8,64E-41 | Com. | 1.05E-38 | Com. | 3,54E-40 |
|  | Indiv. | 1,82E-33 | Indiv. | 2,45E-32 | Village | 4.56E-35 | Village | 1.05E-35 |
|  |  |  |  |  |  |  | Indiv. | 5.46E-34 |

Notes: Model 1 is a multiple logistic regression model; Model 2: is a two-level mixed effect logit model with a random intercept estimate at the survey respondent individual levels; Model 3: is a two-level mixed effect model with a random intercept estimate at the village levels. Model 4: is a two-level mixed effect model with a random intercept estimate at the common levels; Model 5: is a three-level mixed effect model with a random intercept estimate at the village and individual levels. Model 6: is a three-level mixed effect model with a random intercept estimate at the common and individual levels; Model 7: is a three-level mixed effect model with a random intercept estimate at the common and village levels. Model 8: is a four levels mixed effect logit model with random intercept estimate at the common and village levels; BIC: Shwartz-Bayesian Information Criteria; AIC: Akaike Information Criteria; ICC: Intraclass Correlation. **Source : Authors**
