# Supplementary material for: What factors shape an individual’s probability to be enrolled in a professionally-managed community-based health insurance? Results from a cross-sectional case-control study in two districts in Mali
Source: PLOS Glob Public Health. 2025 Jul 8;5(7):e0004892. doi: 10.1371/journal.pgph.0004892 (PMC12237063; doi:10.1371/journal.pgph.0004892)
Supplement: S1 Text — (DOCX) [file pgph.0004892.s002.docx]

Inclusivity in global research

PLOS’ policy on inclusivity in global research aims to improve transparency in the reporting of research performed outside of researchers’ own country or community and ensures that PLOS publications reporting global research adhere to high standards for research ethics and authorship. Authors of relevant research articles may be asked to complete the questionnaire below, which outlines ethical, cultural, and scientific considerations specific to inclusivity in global research. This questionnaire may be requested when researchers have travelled to a different country to conduct research, if research uses samples collected in another country, research with Indigenous populations or their lands, or if research is on cultural artefacts. Researchers travelling to another country solely to use laboratory equipment will not normally be required to complete the questionnaire. However, the questionnaire can be requested at the journal’s discretion for any submission – if you have been requested to complete this questionnaire by the PLOS journal you submitted to, please do so.

Please complete the questionnaire below and include this as a Supporting Information file with your manuscript. Note that if your paper is accepted for publication, this checklist will be published with your article in the supporting information files. Please ensure that you reference the checklist in the main body of your manuscript. We suggest adding a subsection ‘Inclusivity in global research’ to your Methods section and adding the following sentence: “Additional information regarding the ethical, cultural, and scientific considerations specific to inclusivity in global research is included in the Supporting Information (SX Checklist)”

The questions have been designed to be applicable to a wide range of study types, and there are subsections for both human subjects research and non-human subjects research. If any of the questions are not relevant to your research please mark them as “N/A” as appropriate.

**Ethical considerations, permits and authorship**

*This section is applicable to all research types.*

Provide details as to who granted permissions and/or consent for the study to take place in the Methods section of your manuscript. This should include the names of **all** ethics boards, governmental organizations, community leaders or other bodies that provided approval for the study. If individuals provided approval refer to these people by their role or title but do not list their name(s).

Reported on page 14: The Minister of Health and Social Affairs has given its authorization for the collection of information relating to the implementation of this research in 2020, under number 001744 MSAS-SG. The ethics committee of the Institut National de Recherche en Santé Publique (INSRP) approved the protocole.

If there were any deviations from the study protocol after approval was obtained please provide details of these changes in the Methods section of your manuscript.

Reported on pages 15 . 16: With the exception from the change in the process of obtaining individual consent, the protocol was implemented in accordance with its initial plan. In the protocol approved by the ethics committee, written consent was initially required. However, due to the evolving security situation in the field and following the advice of local community leaders, researchers opted for verbal consent. This modification in protocol implementation was submitted to the ethics committee and received its approval. The ethics committee’s approval letter is attached as an appendix to the manuscript.

Did this study involve local collaborators that are residents of the country where the research was conducted or members of the community studied? If you do not have any authors from said communities, please provide an explanation for this below.

Yes, Laurence Touré, Hamidou Niangaly and Dansiné Diarra are reserarchers from Mali. All them are authors in the manuscript.

Everyone listed as an author should meet PLOS’ criteria for authorship and all individuals who meet these criteria should be included in the author byline, rather than the acknowledgements. For further information please see the journal’s Authorship Policy.

**Human subjects research (e.g. health research, medical research, cross-cultural psychology)**

Did you obtain written informed consent from a representative of the local community or region before the research took place? How did you establish who speaks for the community? Details of written informed consent obtained from study participants should be reported separately in the Methods section of your manuscript.

The research protocol has been approved by the ethical comitte of the National Institut of Public Health of Mali. The survey was conducted in insecure areas. To minimize risks for the interviewers, we deemed it preferable to obtain verbal rather than written consent. Displaying paper documents could have drawn the attention of ill-intentioned individuals, potentially exposing the interviewers to the risk of attack. For the same reasons, data collection was carried out using Android phones, which are more discreet than tablets, as their widespread use allows them to go unnoticed. The ethical comitee approved the verbal consent. The approval letter is attached to the manuscript.

How did members of the local community provide input on the aims of the research investigation, its methodology, and its anticipated outcome(s)?

Data collection was conducted through face-to-face interviews using a questionnaire designed in alignment with the research objectives. The questionnaire was developed with the Kobocollect application and installed on mobile devices.

When engaging with the local community, how did you ensure that the informed consent documents and other materials could be understood by local stakeholders?

The survey took place in a secure and confidential setting. Interviews were conducted in French or Bambara, the most widely spoken language in Mali. For participants who did not understand either language, assistance was sought from an individual fluent in one of them, depending on the participant’s native language. All questions were orally translated into Bambara by local interviewers and researchers.

Will the findings of the research be made available in an understandable format to stakeholders in the community where the study was conducted (e.g. via a presentation, summary report, copies of publications, etc.)? Please provide details of how this will be achieved.

A policy note will be prepared to foster the ownership of research findings by the ministries responsible for health and social development. This note will be officially submitted to these ministries.

Additionally, the research findings will be shared at conferences held in Mali, attended by key decision-makers. In this regard, an initial presentation was delivered at the Journées Scientifiques organized by The Francophone Africa & Fragility Network (AFRAFRA) on January 7-8, 2025, in Bamako, with the participation of technical advisors from the Ministry of Health and the Caisse Nationale d'Assurance Maladie.

**Non-human subjects research using specimens/ animals collected as part of the study, or those housed in archival collections. Examples include archaeology, paleontology, botany and zoology.**

Did the permission you obtained from a local authority to perform the study include an agreement on access to outputs and benefit sharing? This may include procedures to enable fair distribution of the benefits and resources arising from the research performed. Please include any details of Prior Informed Consent and Benefit Sharing Agreements obtained. These may be required by field-specific regulations, for example the Convention on Biological Diversity (CBD) and the associated Nagoya Protocol.

N/A

If the material used in your study was imported, please A) provide the year it was imported and B) indicate whether permits were obtained to import/export the materials used, C) provide details of any permits obtained. If this information is not available, please indicate this.

N/A

If you used archival specimens, please state how the material used in your study was acquired by the institute it is held in and provide details of any permits obtained for the original excavations/ sample collection. If this information is not available, please indicate this.

N/A

How was the potential cultural significance of the materials collected in your study to local communities considered in your research design? Were Indigenous peoples and/or local researchers and institutions involved with archaeological excavations / collection of specimens? If so, please provide a description of their involvement.

N/A

If your manuscript includes photographs of human remains please indicate whether authors obtained permission from descendants or affiliated cultural communities to do so.

N/A
