# Supplementary material for: What factors shape an individual’s probability to be enrolled in a professionally-managed community-based health insurance? Results from a cross-sectional case-control study in two districts in Mali
Source: PLOS Glob Public Health. 2025 Jul 8;5(7):e0004892. doi: 10.1371/journal.pgph.0004892 (PMC12237063; doi:10.1371/journal.pgph.0004892)
Supplement: Attachment — Submitted filename: PGPH-D-24-02388.pdf [file pgph.0004892.s003.pdf]

PLOS Global Public Health

What factors shape an individual’s probability to be enrolled in a professionally-managed Mutuelle? Results from a cross-sectional case-control study in two districts in Mali

--Manuscript Draft--

**Manuscript Number:**	PGPH-D-24-02388
**Article Type:**	Research Article
**Full Title:**	What factors shape an individual’s probability to be enrolled in a professionally-managed Mutuelle? Results from a cross-sectional case-control study in two districts in Mali
**Short Title:**	Results from a cross-sectional case-control study in two districts in Mali
**Corresponding Author:**	Hamidou Niangaly, PhD<br>National Institute of Public Health<br>Bamako, MALI
**Order of Authors:**	Hamidou Niangaly, PhD
	Manuela De Allegri, PhD
	Laurence Touré
	Dansiné Diarra, PhD
	Valéry Ridde, PhD
**Keywords:**	Mutuelle; Health; Professional
**Abstract:**	Introduction<br>Across West Africa, countries are promoting new models of Community-based health insurance to overcome the challenges faced by existing schemes. We investigated factors associated with an individual’s probability to be enrolled in one of two professionally-managed Mutuelles in Mali.<br>Methods<br>We carried out a cross-sectional survey adopting a case-control methodology and used multi-stage sampling to select Mutuelle members and non-members. The primary outcome variable was being enrolled or not in the Mutuelle. Three-level mixed-effects logistic regression model was used to estimate the association between individual enrolment and a series of individual, household, and wider community factors.<br>Results<br>Of the 1,270 people surveyed 847 and 423 were non-members and members respectively. Respondents with primary education (AOR = 1.6, P = 0,035), secondary education (AOR = 2.31, P = 0,001) or higher school/University (AOR = 5.5, P < 0,001); who were economically active (AOR = 1.52, P = 0,05) and considered themselves wealthy (AOR = 1.94, P < 0,001) were more likely to subscribe to Mutuelles. Those who reported having a good perception of their health (AOR = 0.68, P = 0,038) were less likely to subscribe to Mutuelles.<br>Conclusion<br>Albeit substantially higher than what experienced by community-based schemes, membership in these professionally-managed Mutuelles remains low and the determinants of participation do not differ substantially from what reported in the general literature. Targeting the needs of poor vulnerable people and more pervasive communication strategies are urgently needed to enhance enrolment in these new Mutuelles and ensure the way to UHC.
**Additional Information:**	
**Question**	**Response**
**Financial Disclosure**	This research was funded by Agence Française de Développement.

Powered by Editorial Manager® and ProduXion Manager® from Aries Systems Corporation

Enter a financial disclosure statement that describes the sources of funding for the work included in this submission and the role the funder(s) played. This includes grants and any commercial funding of the work or authors.<br><br>This statement will be typeset if the manuscript is accepted for publication.<br><br>*Please review the submission guidelines and the instructions link below for detailed requirements and guidance.*	
**Competing Interests**<br><br>On behalf of all authors, disclose any competing interests that could be perceived to bias this work.<br><br>This statement will be typeset if the manuscript is accepted for publication.<br><br>*Please review the instructions link below and PLOS Global Public Health's competing interests policy to determine what information must be disclosed at submission.*	Authors declare no competing interests
**Data Availability**<br><br>Before publication, Authors are required to make fully available and without restriction all data underlying their findings. Please see our PLOS Data Policy page for detailed information on this policy.<br><br>A **Data Availability Statement**, detailing where the data can be accessed, is required at first submission. Insert your Data Availability Statement in the box below.	Data will be available at request.

Please see the data reporting section of our submission guidelines for instructions on what you need to include in your Data Availability Statement.<br><br>This statement will be typeset if the manuscript is accepted for publication.<br><br>*PLOS allows rare exemptions to address legal and ethical concerns. If you have legal or ethical restrictions, please use the box below to detail these in full sentences for the Journal team to consider.*	

Powered by Editorial Manager® and ProduXion Manager® from Aries Systems Corporation

Manuscript

Click here to access/download;Manuscript;Manuscrit - Factors associated to enrolment in CBHI -Mali.docx

Title: What factors shape an individual's probability to be enrolled in a professionally-managed Mutuelle? Results from a cross-sectional case-control study in two districts in Mali

Hamidou Niangaly [1*], Manuela De Allegri[2], Laurence Touré [3], Dansiné Diarra[4] and Valéry Ridde[5,6]

[1] Institut National de Santé Publique; hamidouniangaly@gmail.com

[2] Heidelberberg Institute of Global Health, Heidelberg University Hospital and Faculty of Medicine, Heidelberg University, Germany; manuela.deallegri@uni-heidelberg.de

[3] Miseli; chat@afribonemali.net

[4] Université des Sciences Sociales et de Gestion de Bamako; dansined@gmail.com

[5] Institut de Santé et Développement, Université Cheikh Anta Diop, Dakar, Sénégal; valery.ridde@ird.fr

[6] Université Paris Cité, IRD, Inserm, Ceped, 45, Rue des Saints Pères, Paris F-75006, France ; valery.ridde@ird.fr

***Correspondence**: hamidouniangaly@gmail.com (HN)

<u>Abstracts</u>

Introduction

Across West Africa, countries are promoting new models of Community-based health insurance to overcome the challenges faced by existing schemes. We investigated factors associated with an individual's probability to be enrolled in one of two professionally-managed Mutuelles in Mali.

Methods

We carried out a cross-sectional survey adopting a case-control methodology and used multi-stage sampling to select Mutuelle members and non-members. The primary outcome variable was being enrolled or not in the Mutuelle. Three-level mixed-effects logistic regression model was used to estimate the association between individual enrolment and a series of individual, household, and wider community factors.

Results

Of the 1,270 people surveyed 847 and 423 were non-members and members respectively. Respondents with primary education (AOR = 1.6, P = 0,035), secondary education (AOR = 2.31, P = 0,001) or higher school/University (AOR = 5.5, P < 0,001); who were economically active (AOR = 1.52, P = 0,05) and considered themselves wealthy (AOR = 1.94, P < 0,001) were more likely to subscribe to Mutuelles. Those who reported having a good perception of their health (AOR = 0.68, P = 0,038) were less likely to subscribe to Mutuelles.

Conclusion

Albeit substantially higher than what experienced by community-based schemes, membership in these professionally-managed Mutuelles remains low and the determinants of participation do not differ substantially from what reported in the general literature. Targeting the needs of poor vulnerable people and more pervasive communication strategies are urgently needed to enhance enrolment in these new Mutuelles and ensure the way to UHC.

Keywords: *Mutuelle*; Health; Professional

Introduction

Community-based health insurance (CBHI) operates voluntarily, is managed by volunteers, and is characterized by community members pooling funds to offset healthcare costs (1). In West Africa, CBHI schemes, usually referred to as *Mutuelles*, are traditionally set up on a small scale, so that risk sharing is confined to the municipality level (2). Since the 1980s, these *Mutuelles* have been established across West Africa with the help of technical and financial partners, such as World Bank and U.S. Agency for International Development (3). However, despite this external support, these *Mutuelles* consistently suffered from low penetration rates and never managed to achieve high population coverage (4). Many studies carried out in the 1990s and 2000s tried to understand factors associated with population acceptance to support projects and States investing in CBHI as a temporary instrument towards fostering Universal Health Coverage (UHC) (4–7). The main factors identified to explain low participation in small-scale volunteer-run *Mutuelles* are: low contributory capacity of villagers, lack of trust in the management structures, poor perceptions of quality of care provided, limited provision of services, the persistence of co-payments and the periodicity of contributions, previous negative experiences with community associations (6–9).

In the early 2020s, in light of persisting low penetration rates and in light of the criticism by the World Health Organization (WHO), clearly pointing out at the system weaknesses, some countries in the region returned to a suggestion once advanced by Bart Criel (10) in the 1990s and proposed to expand the *Mutuelle* risk pooling at the district level. For instance, Mali introduced two pilot

Mutuelles managed at district level and by professionals (11,12). Details of these schemes' implementation have been described before (11,12). In 2018, less than 3% of Mali's population was covered by a CBHI (11), but these two *Mutuelles* alone managed to cover 9% of the population in less than three years of operation. While this success is noteworthy, it remains relatively low considering overall coverage needs.

To support Malian officials looking for solutions to expand UHC, it is important to understand what factors contributed to people's decision to join this new type of *Mutuelle*. To our knowledge, no other study in West Africa has explored determinants of participation in CBHI managed by professionals and organized at a district level, even in Senegal where they have been in place in two departments since 2014 (13). This is important since it represents a fundamental departure from the traditional CBHI management model, requiring people to trust a much larger structure.

We aimed to fill this gap in knowledge by examining what factors shape participation to district and professionally-run *Mutuelles* in Mali. Our ultimate objective is ultimately to inform public officials in their decision-making towards UHC.

Materials and Methods

Study site

As part of the Health and Social Development Support Programme (PADSS2) in the Mopti Region, two professionally managed *Mutuelles* have been implemented in the administrative circles (districts) of Mopti and Bandiagara. The cercles of Mopti and Bandiagara are located in central Mali,

in the Mopti region, with the populations of 492 970 and 418 873 inhabitants in 2018 respectively according to estimates by the National Population Direction.

Study period

The study was carried out over 6 months, including the preparatory phase, data collection and report writing. Data collection took place from June 2 to August 8, 2022.

Study population

The study population was the target population of *Mutuelles* which represents 78% of the population of the Mopti and Bandiagara cercles. This population is made up of workers in the informal sector which is neither covered by the compulsory health insurance reserved for employees and civil servants nor by the medical assistance scheme intended solely for indigent persons as defined by the social services administration. We restricted our study to persons aged 18 and older.

Management of the Mutuelles

The two *Mutuelles* are managed by agents trained in the implementation of a mutualist system through the technical support of the Union technique de la mutualité malienne and receive financial support from the Programme de santé et de développement social. Elected mutualists were elected in each **municipality** to better communicate about this innovation in the villages. Employee managers were recruited according to the size of the municipality (one manager for two or three

municipalities). Their role was to communicate about ***Mutuelles*** societies and their products, but also to monitor membership and collect contributions. This professional management thus makes these ***Mutuelles*** different from those experienced in previous research in Mali and elsewhere (13–15).

Sampling

We relied on a three-stage random sampling to select municipalities, villages, and members of the *Mutuelles* in each cercle. As described hereafter, we combined random and purposive sampling technique to select the communities where to conduct our survey. This choice was imposed by security considerations. We defined as cases individual who had paid their full membership fees and for whom the insurance coverage had taken effect, regardless of the status of their contribution (updated or not). We defined as controls all individuals without a *Mutuelle* membership.

Sample sizes and selection procedure

Municipalities

The municipality is an administrative division that groups together several villages. Due to insecurity, we first drew a list of accessible areas in agreement with village, political, and local administrative authorities. Out of 15 municipalities in the circle of Mopti, three municipalities were accessible (20%): Socoura, Mopti, and Sio. In the Bandiagara circle, 10 out of 21 municipalities were accessible (47.6%): Sangha, Kendié, Wadouba, Soroly, Bandiagara, Dandoli, Dourou, Diamnati, Pelou, and Ségué Iré. The survey was carried out in the three accessible municipalities of Mopti and

the 10 accessible municipalities of Bandiagara, i.e., on average 36.1% of the municipalities of the two circles.

Villages

From all villages included in the accessible municipalities, we further excluded villages with five or fewer members, due to feasibility concerns. This resulted in a total sampling frame of 155 villages, 124 and 31 in Bandiagara and Mopti respectively. From this total of 155 villages, we selected 49 villages, 37 in Bandiagara and 9 in Mopti (sampling proportional to circle size) to be included in our survey.

Members and non-members

To identify respondents to include in our study, we relied on the single population proportion formula (16) and adopted the proportion of two controls (non-*Mutuelle* members) to one case (*Mutuelle* member). The formula below (Equation 1) was used to calculate the sample size for each group (cases and controls):

$$n = z^2 * \frac{pq}{d^2} \quad (1)$$

Where p = 1-q, z is the value dependent on the chosen error risk α (z = 1.96 for α=5%); d, the desired precision, and n = size of the sample. For an average penetration rate of the *Mutuelle* in the Mopti and Bandiagara cercles in December 2021 of 9.1% (n/N = 12619/138670) which corresponds to the value q, 1-q = 0.91, a desired precision of 3%, a confidence level of 95%, a design effect of 1.5 and a

non-response rate of 10%, a total size of 1270, i.e., 423 members and 847 non-members was determined to be sufficient to identify factors associated with membership in the *Mutuelle*.

Recruitment of members (cases)

We aimed at producing a sample of *Mutuelle* members proportional to number of members in each village. We could rely on official membership lists to guide our sampling and drew our samples at random from these lists. However, due to security reasons, we could not always locate the households we had identified and had to adjust sampling to the reality of the fact that some members had relocated due to insecurity concerns.

Recruitment of non-members (witnesses)

Given the absence of adequate census data from which to draw the control sample, we had to rely on pragmatic techniques to identify controls. Hence, as first control, we selected the third compound to the East of the *Mutuelle* member respondent and as second control, we selected the third compound to the East of the latter. In case non suitable compound could be identified in the Eastward direction, the investigator turned South to identify an appropriate control compound.

Once in a compound, controls were selected at the individual level to match the Mutuelle member case's sex and status within the household (i.e., household head, spouse, son, or daughter). Moreover, in the case of multi-household compound, households were selected to match the same household status as the corresponding member household (e.g., dominant versus subordinated household).

Variables and their measurement

Dependent variable

The dependent variable was defined as membership in the *Mutuelle*. It was specified as equal to 1 if the person was enrolled, and as equal to 0 if not. As mentioned earlier, membership was defined as having joined the scheme at any one point in time, with no actual checks of whether the member was up to date with the payment of the membership fee. Members will have all started to benefit from insurance coverage.

Independent variables

The independent variables selected were those previously found to be associated with membership in CBHI (17–20). Independent variables at the individual level measured the socio-demographic profile of members and non-members. They included sex, age, level of education, participation in economic activity, membership status in a formal organization, wealth status, perceived health status, whether or not chronic illness was present, whether or not a disability was present, and the source of information on the existence of a *Mutuelle* in the locality. Independent variables at the household level included socio-economic status, derived combining household material assets using principal component analysis. Notably, the main component of the polychoric correlation matrix (21,22) was used to determine the household wealth index. Following the guidelines of the Demographic and Health Survey, wealth quintiles were created by ordering the wealth index into

five equal parts (23). Households were accordingly classified as very poor, poor, intermediate, less poor, and least poor. At village level, we differentiated urban and rural localities.

Empirical approach

Given our binary dependent variable, we adopted a logistic regression model. Since individuals were nested in villages, the assumption of independence inherent of standard logistic regression was violated and we had to rely on a multi-level model, to take into account the hierarchical structure of our data and the resulting intra-cluster variability. Following Bekele 2022, we used a three-level mixed-effects logistic regression model to determine the factors that influence decision to join or not to join enrolment in the M*utuell*e membership (15). We modeled the probability of being registered in the *Mutuelle* using a three-level model, estimating the equations ((2) and (3)) below:

$$log\left(\frac{\pi_{ij}}{1-\pi_{ij}}\right) = \beta_0 + \beta_1 X_{ij} + \beta_2 F_{ij} + \mu_j (2)$$

$$\mu_j \sim \left(\sigma_\mu^2\right) (3)$$

Where i and j are the units of level 1 (individuals = survey respondents) and level 3 (village and municipality), respectively; X and F apply to the variables of the level of the individual and the municipality and village respectively, π_{ij} is the probability of enlisting in the *Mutuelle* in the community for the third individual; β are the fixed coefficients-therefore, there is a corresponding efficiency for each increase of a unit of X|F (a set of predictors). Whereas in the absence of control of predictors, β0 is the intercept, capturing the effect on the probability of the individual to subscribe

to the *Mutuelle*; and μ_j indicates the random effect for the municipality and village (effect of the municipality or village on the individual's decision to subscribe to the *Mutuelle*). The existence of cluster data and variations within and between communities (municipality and village) were taken into account assuming that each community had a different intercept (β0) and fixed coefficient (β). The log-likelihood ratio (LLR), Akaike Information Criteria (AIC) and Schwartz-Bayesian Information Criteria (BIC) tests were used to evaluate the best fit model (24). The Wald test was used to investigate the random effects at village and municipality- levels (25). The importance of incorporating random parameter estimates was investigated using intraclass correlation test (24,26). The near-zero intraclass correlation test (ICC) values indicate that the characteristics of persons surveyed within municipalities or villages are no more similar than those of persons surveyed across different municipalities or villages. More details on tests are given in appendix section. Data have been analyzed using Stata software (15) (Stata Statistical Software: Release 14. College Station, TX: StataCorp LP). The *melogit* code has been used to fit mixed logit model. Appropriate statistical tests (Student, Pearson) were used to measure associations with an alpha risk of 5%.

Ethical clearance

This study is part of an extensive research program (Couverture Universelle Santé au SAHEL), which was approved by the institutional ethics committee of the National Institute of Public Health of Mali, on December 26, 2018, decision no. 34/2018/CE-INRSP). Verbal consent was obtained from all participants prior to data collection. We asked permission from community leaders and local authorities. Prior to the start of data collection, the delegates from the community-based health

insurance scheme informed community leaders and local government representatives of the research. We sought community permission from customary leaders such as village chiefs. In each village, the investigators visited the village chief to explain the purpose and method of the research. The authorization was obtained from local health officials and government representatives before the survey.

Protocol Deviation

The protocol was carried out according to plan. There was no violation of the protocol.

Inclusivity in global research

Additional information regarding the ethical, cultural, and scientific considerations specific to inclusivity in global research is included in the Supporting Information (SX Checklist).

Results

Characteristics of the persons surveyed

Table 1 provides a summary of the characteristics of members and non-members. A total of 1,270 people were surveyed, including 847 non-members and 423 members. There were more men than women among both members and non-members, with 70.8% (600/847) and 72% (303/423) respectively. Large proportions of non-members (47.7%) and members (32.4%) were out of school, $P < 0.001$. Among those who attended school, with the exception of primary level, where there was no statistically significant difference between the two comparison groups, the proportion of respondents who had reached secondary school and university levels was higher among members

than non-members, P < 0.05. As regards participation in economic activities, there were more economically active people among members (86.5%) than among non-members (78%), P = 0.001. Respondents who were members of a formal organization were more likely to be members (24.8%) of a *Mutuelle* than non-members (13.7%), P = 0.001. Those who considered themselves wealthy were also more numerous among members (25.3%) than among non-members (11.8%), P = 0.001. The percentage of respondents with chronic illness was higher among members (17%) than among non-members (12.3%). When looking at the household wealth quintile, 47.5% of members and 36.3% of non-members were in less/least poor households, P = 0.001. The two groups were statistically comparable in terms of disability, those who considered themselves in good health and sources of information (radio, television, village authorities) and location (rural or urban)

Table 1: Characteristics of survey participants

		No-member		Member		
Variables	Categories	Freq.	%	Freq.	%	P-value
Gender						
	Female	247	29,2	120	28,4	
	Male	600	70,8	303	71,6	0,77
Education level						
	Education No	404	47,7	137	32,4	< 0.001
	Primary	111	13,1	54	12,8	

	Secondary	72	8,5	55	13	
	College/University	59	7	93	22	
	Koranic	201	23,7	84	19,9	
Economically active						
	No	186	22	57	13,5	< 0.001
	Yes	661	78	365	86,5	
Member of formal organization						
	No	731	86,3	318	75,2	< 0.001
	Yes	116	13,7	105	24,8	
Consider yourself rich						
	No	747	88,2	316	74,7	< 0.001
	Yes	100	11,8	107	25,3	
Has disability						
	No	816	96,3	406	96	
	Yes	31	3.7	17	4	
Has Chronic disease						
	No	743	87,7	351	83	0,021
	Yes	104	12,3	72	17	
Self-reported health status						
	Bad	191	22,6	106	25,1	0,319
	Good	656	77,4	317	74,9	

Household wealth						
	Poorest	187	22,1	67	15,8	< 0.001
	Poor	199	23,4	57	13,5	
	Middle	154	18,2	98	23,2	
	Less poor	164	19,4	90	21,3	
	Least poor	143	16,9	111	26,2	
Source of information on the *Mutuelle*						
Radio						
	No	388	70,8	309	73	0,44
	Yes	160	29,2	114	27	
TV						
	No	499	91,1	372	87,9	0,11
	Yes	49	8,9	51	12,1	
Village authorities						
	No	487	88,9	371	87,7	0,58
	Yes	61	11,1	52	12,3	
Location						
	Rural	553	65,3	276	65,2	0,99
	Urban	294	34,7	147	34,8	

Source : Authors

Factors associated with subscription to new Mutuelle

Predictors at the individual level

Table 2 presents estimates using the three-tiered mixed-effects logistic regression method. Columns (1), (2), and (3) contain Odds gross ratios (CORs), confidence interval values (IC95%), and probability values (P-value). Columns (4), (5), and (6) contain Odds adjusted ratios (AORs), confidence interval values (IC95%), and probability values (P-value). Respondents' level of education, participation in economic activity, perceived wealth, perception of good health, and household size were significant predictors of new *Mutuelles*. Compared to respondents who were not in school, those who had reached primary level (between 1 and 6 years of education), second cycle level (between 7 and 9 years of study) and high school or university level (10 years of study or more) were respectively 1.6 times (AOR = 1.6; IC = 1.03-2.48; P = 0.035), 2.31 times (AOR = 2.31; IC95% = 1.43-3.71; P = 0.001) and 5.5 times (AOR = 5.5; IC95% = 3.31-9.14; P < 0.001) more likely to subscribe to the *Mutuelles*. Economically active respondents or those who considered themselves wealthy were 1.52 times (AOR = 1.52; CI95% = 1-2.31; P = 0.05) and 1.94 times (AOR = 1.94; CI95% = 1.35-2.79; P < 0.001) more likely to subscribe to the *Mutuelles* than those who had no activity or those who declared to be poor. Regarding perceived health status, respondents who reported being in good health were 0.68 times (AOR = 0.68; CI 95% = 0.47-0.98; P = 0.038) less likely to subscribe to the *Mutuelles* than those who felt in poor health. The probability of subscription to the *Mutuelles* was 1.07 times higher among households with a large number of people than those with few people (AOR = 1.07; CI 95% = 1.02-1,11; P = 0.003).

Predictors at the Community level

The only variable that characterized the community level in our study was the level of development of the villages where the respondents lived (rural or urban). Respondents who lived in rural areas had the same level of subscription to the *Mutuelles* as those living in urban areas (AOR = 1.04; CI 95% = 0.75-1.46, P = 0.8).

Random effects variation measures

Estimates of random effects at the municipality (Variance = 3.44x10 $^{-38}$; SE = 2.85 10^{-21}) and village (Variance = 1.5 x10-34; SE = 2,21x10^{-18}) levels show very low variances. This suggests that municipalities and villages were similar. None of the model fit tests (AIC, BIC, LLR) discriminates between the different models. The Wald test statistics at the municipality (Wald statistics = 1.47x10^{-34}, P > 0.05) and the village (Wald statistics = 4.6x10^{-33}, P > 0.05) levels were not statistically different from zero. Based on the results of the Wald test, we cannot conclude to the presence of random effects at the supra-individual level (municipalities and villages) that influence the decision to enter in the *Mutuelle*. However, given the hierarchical structure of our data, the three-tiered mixed effects logistics model was used as appropriate to determine predictive factors for *Mutuelle* subscription.

Table 2: *Factors associated with enrollment in the Mutuelle*

		(1)	(2)	(3)	(4)	(5)	(6)
Variables	Categories	COR	CI95 %	P-value	AOR	CI95 %	P-value
Gender	Male	Ref.			Ref.		
	Female	1,04	0,80-1,35	0,769	0,83	0,59-1,18	0,299

Age		0,99	0,99-1,00	0,139	1	0,98-1,01	0,588
Education level	Education No	Ref.			Ref.		
	Primary	1,43	0,98-2,09	0,062	1,60	1,03-2,48	0,035
	Secondary	2,25	1,51-3,36	< 0.001	2,31	1,43-3,71	0,001
	College/University	4,65	3,18-6,79	< 0.001	5,50	3,31-9,14	< 0.001
	Koranic	1,23	0,89-1,70	0,201	1,36	0,93-1,98	0,11
Economically active	No	Ref.			Ref.		
	Yes	1,80	1,30-2,49	< 0.001	1,52	1,00-2,31	0,05
Member of formal organization	No				Ref.		
	Yes	2,08	1,55-2,79	< 0.001	1,3	0,92-1,84	0,142
Consider yourself rich	No	1	1,00-1,00		Ref.		

	Yes	2,53	1,87-3,42	< 0.001	1,94	1,35-2,79	< 0.001
Has chronic disease	No				Ref.		
	Yes	1,47	1,06-2,03	0,022	1,43	0,93-2,19	0,1
Has disability	No				Ref.		
	Yes	1,1	0,60-2,02	0,752	1,18	0,58-2,40	0,652
Self-reported health status	Bad	1	1,00-1,00		Ref.		
	Good	0,87	0,66-1,14	0,32	0,68	0,47-0,98	0,038
Household size		1,06	1,03-1,09	< 0.001	1,07	1,02-1,11	0,003
Household wealth quintile	Poorest	1	1,00-1,00		Ref.		
	Poor	0,8	0,53-1,20	0,28	1,03	0,65-1,64	0,899
	Middle	1,78	1,22-2,59	0,003	1,4	0,90-2,20	0,138

	Less poor	1,53	1,05-2,24	0,028	1,35	0,87-2,12	0,185
	Least poor	2,17	1,49-3,15	< 0.001	1,49	0,94-2,36	0,086
Source of information							
Radio	No	Ref.					
	Yes	0,89	0,67-1,19	0,441	0,75	0,54-1,04	0,085
TV	No	Ref.					
	Yes	1,4	0,92-2,11	0,115	1,33	0,82-2,15	0,253
Village authorities	No	Ref.					
	Yes	1,12	0,75-1,66	0,576	1,33	0,86-2,08	0,202
Rental	Rural	1	1,00-1,00		Ref.		
	Urban	1	0,78-1,28	0,988	1,04	0,75-1,46	0,8
Random intercepts							
					Variance	SE	IC95%

	Municipality				3.44E-38	2,85E-21	—
	Village				1.50E-34	2,21E-18	—
Number of observations					970		
Model statistics - values							
	Chi 2				106,68		
	BIC				1352,89		
	AIC				1245,59		
Log Likelihood					—600,79		
ICC					ICC	SE	IC95 %
	Municipality				1.05E-38	8,67E-22	—
	Village				4.56E-35	6,73E-19	—

Notes: Columns (1), (2) and (3) contain the crude odds ratio (COR), Interval confidence at 95% (IC95%) and the values of the probability (P-value). Columns (4), (5), and (6) contain the adjusted odds ratio (AOR), Interval confidence at 95% (IC95%), and the values of the probability (P-value); Chi 2 is the model fitness test under the Chi-squared distribution, BIC = Shwartz-Bayesian Information Criteria, AIC = Akaike Information Criteria, ICC = Intra Class Correlation. **Source : Authors**

Discussion

This study makes a unique contribution to the literature by being one of the first studies examining determinants of participation to the new professionalized *Mutuelles,* within the context of the newly restructured mutualist system in two administrative cercles (Mopti and Bandiagara) in Mali.

Despite the professionalization of the *Mutuelles,* the beneficiary coverage rate remained low (9%) in the context of this project. This low level of membership is reminiscent of the difficulty of Community health insurance in reaching the expected coverage of their target population (27). Our study reveals that factors associated with participation in a professional *Mutuelle* do not differ from factors associated with participation in traditional volunteer-based *mutuelles* (8,9,28–31). Thus, despite the relative effectiveness of the project, which has been able, in less than three years, to reach a penetration rate never achieved before, the same set of socio-demographic and economic factors continue to explain who is covered and who is not covered.

Factors capturing an individual socio-economic status (level of education, perception of one's wealth status and ability to pay; household size, and perceived health status) were all found to be significantly associated with one's probability of being enrolled in the *Mutuelle*. Our findings on the effect of socio-economic status resonate with what has been observed in other settings, such as Mali, Senegal, Ghana and Asia countries (8,9,28–31). This is not surprising since economically active and better-off individuals have a higher purchasing power and therefore enjoy a higher ability to pay the scheme premiums. It is important to recall that the State of Mali, aware of the challenges of membership, subsidizes the rate of contributions to all *Mutuelles* in the country up to 50%, as in Senegal (32). These two professionals Mutuelles received an additional 30% subsidy, funded by donors. This 80% subsidy of the real premium probably explains the relative success observed in terms of enrollment, reaching 9%, but leaves open questions in terms of the equity of the subsidization, since it appears that poorer individuals were still less likely than least-poor individuals to enroll. It needs to be noted here that in principle, the ultra-poor (*indigents*) are entitled

to receive services completely free of charge thanks to the RAMED policy (33,34). Still, not only is implementation of the policy far from being effective, but the proportion of individuals covered is too small compared to the actual inability to join the scheme observed in our study. A similar situation is also observed in Senegal where the *indigents* have a right to join the *Mutuelles* free of charge within the framework of a national exemption policy, but do not do so due to lack of information and resources (35,36).

The relationship between higher educational and enrollment in CBHI has been observed before in settings such as Burkina Faso and Ethiopia countries (7,18,20) and can be explained by the fact that higher educational levels increase awareness of risk minimization strategies and hence decision to join health insurance schemes. The fact that we observe an effect of education, independently of socio-economic status, calls into question promotion activities, clearly pointing at the need for more targeted information and communication campaigns.

In line with prior studies, we also observed that individuals who perceived themselves to have a higher health status were less likely to join the *Mutuelle* (37). This is likely to be an indication of adverse selection, and represents a risk for the long-term financial viability of the scheme. The challenge the *Mutuelles* face is to change risk perceptions among the target populations to attract a wider constituency of beneficiaries and by doing so, enhancing the financial viability of the scheme.

Conclusions

Despite the challenges of operating in a setting affecting by insecurity, this study managed to rely on a rigorous methodology to assess factors associated with adherence to the innovative CBHI

model of the professional *Mutuelles* in Mali. Two years into their implementation, these *Mutuelles* reached 9% of the target population.

While our study was not set to assess the effectiveness of the project, it allowed us to investigate what challenges to membership persisted. Considering the persistence of important socio-economic and educational inequities in individual enrollment, group enrollment strategies (cooperatives, schools, villages; etc.) could be tested as a viable option to overcome existing inequities. Moreover, it is fundamental to ensure that the ultra-poor are included through the systematic integration of targeted exemption policies in emerging insurance schemes. Last, but surely not least, the urgency remains to ensure access to quality health care in a country and region that faces unprecedented security challenges.

Funding

This research was funded by *Agence Française de Développement*.

Institutional Review Board Statement

The Ethics Committee authorized this research by Decision No. 34/2018/CE-INRSP of 26 December 2018. All individuals were informed of the ethical issues and were allowed to withdraw from the study at any time. They all agreed to participate through verbal informed consent.

Informed Consent Statement

Informed consent was obtained from all subjects involved in the study.

Acknowledgments

Thank you to all participants in the survey. Our deep gratitude to the investigators who braved difficult conditions on the ground, including insecurity to gather quality information.

Conflicts of Interest

The authors declare no conflicts of interest

References

1. Bennett S. The role of community-based health insurance within the health care financing system: a framework for analysis. Health Policy Plan [Internet]. 2004 May 1 [cited 2023 Oct 21];19(3):147–58. Available from: https://dx.doi.org/10.1093/heapol/czh018
2. Criel B, Atim C, Basaza R, Blaise P, Waelkens MP. Editorial: Community health insurance (CHI) in sub-Saharan Africa: researching the context. Tropical Medicine & International Health [Internet]. 2004 Oct 1 [cited 2023 Oct 21];9(10):1041–3. Available from: https://onlinelibrary.wiley.com/doi/full/10.1111/j.1365-3156.2004.01315.x
3. Ridde V, Caffin JH, Hane F. External influences over Senegalese health financing policy: delaying universal health coverage? Health Policy Plan [Internet]. [cited 2023 Dec 8]; Available from: https://dx.doi.org/10.1093/heapol/czad108
4. Waelkens MP, Werner S, Bart C. Community Health Insurance in Low- and Middle-Income Countries. International Encyclopedia of Public Health. 2017 Jan 1;82–92.

5. Dror DM, Shahed Hossain SA, Majumdar A, Koehlmoos TLP, John D, Panda PK. What factors affect voluntary uptake of community-based health insurance schemes in low- and middle-income countries? A systematic review and meta-analysis. PLoS One. 2016;11(8):1–31.
6. Adebayo EF, Uthman OA, Wiysonge CS, Stern EA, Lamont KT, Ataguba JE. A systematic review of factors that affect uptake of community-based health insurance in low-income and middle-income countries. BMC Health Serv Res [Internet]. 2015 Dec 8 [cited 2023 Oct 28];15(1):1–13. Available from: https://bmchealthservres.biomedcentral.com/articles/10.1186/s12913-015-1179-3
7. Manuela De Allegri, a, Bocar Kouyaté, b, Heiko Becher, a, et al. Understanding enrolment in community health insurance in sub-Saharan Africa: a population-based case–control study in rural Burkina Faso. Bull World Health Organ [Internet]. 2006;84(11). Available from: http://www.who.int/bulletin/volumes/84/11/06-031336.pdf
8. Waelkens MP, Werner S, Bart C. Community Health Insurance in Low- and Middle-Income Countries. International Encyclopedia of Public Health [Internet]. 2017 Oct 6 [cited 2023 Oct 21];82–92. Available from: http://hdl.handle.net/2013/
9. Dror DM, Shahed Hossain SA, Majumdar A, Koehlmoos TLP, John D, Panda PK. What Factors Affect Voluntary Uptake of Community-Based Health Insurance Schemes in Low- and Middle-Income Countries? A Systematic Review and Meta-Analysis. PLoS One [Internet]. 2016 Aug 1 [cited 2023 Apr 7];11(8). Available from: /pmc/articles/PMC5006971/
10. Criel B. District-based health insurance in sub-Saharan Africa. Part I: from theory to practice. Stud Hlth Serv Pol. 1998 Oct;9.
11. Boivin PRADDSFDSDYTLRV. Acceptabilité d'un dispositif de mutuelles de santé innovant au centre du Mali : une recherche en méthodes mixtes [Internet]. 2023 [cited 2023 Oct 22]. Available from: https://zenodo.org/records/8414157
12. Touré L, Boivin P, Diarra Y, Diabaté S, Ridde V. Innovations in mutuality: challenges and learnings for the Universal Health Insurance Plan in Mali. BMJ Glob Health [Internet]. 2023 [cited 2023 Oct 28];7:11055. Available from: http://gh.bmj.com/
13. Ridde V, Ba MF, Guyot M, Kane B, Mbow NB, Senghor I, et al. Factors that foster and challenge the sustainability of departmental health insurance units in Senegal. Int Soc Secur Rev. 2022 Apr 1;75(2):97–117.
14. Abraham E, Gray C, Fagbamigbe A, Tediosi F, Otesinky B, Haafkens J, et al. Barriers and facilitators to health insurance enrolment among people working in the informal sector in Morogoro, Tanzania. AAS Open Res. 2021;4:45.
15. Bekele WT. Predictors of Community-Based Health Insurance in Ethiopia via Multilevel Mixed-Effects Modelling: Evidence from the 2019 Ethiopia Mini Demography and Health Survey. ClinicoEconomics and Outcomes Research. 2022;14(August):547–62.
16. Geferso AT, Sharo SB. Community-Based Health Insurance Utilization and Its Associated Factors among Rural Households in Akaki District, Oromia, Ethiopia, 2021. Adv Public Health. 2022;2022.
17. Conde KK, Camara AM, Jallal M, Khalis M, Zbiri S, De Brouwere V. Factors determining membership in community-based health insurance in West Africa: a scoping review. Glob Health Res Policy [Internet]. 2022;7(1). Available from: https://doi.org/10.1186/s41256-022-00278-8

18. Desalegn M. Determinants of Enrolment in Community Based Health Insurance program among households in East Wollega Zone , west Ethiopia : Unmatched Case-Control Study. 2022;
19. Mirach TH, Demissie GD, Biks GA. Determinants of community-based health insurance implementation in west Gojjam zone, Northwest Ethiopia: A community based cross sectional study design. BMC Health Serv Res. 2019;19(1):1–8.
20. Nageso D, Tefera K, Gutema K. Enrollment in community based health insurance program and the associated factors among households in Boricha district, Sidama Zone, Southern Ethiopia; a cross-sectional study. PLoS One [Internet]. 2020;15(6):1–14. Available from: http://dx.doi.org/10.1371/journal.pone.0234028
21. Uebersax JS. The_Tetrachoric_and_Polychoric_Correlati(3). 2015;1–15.
22. Martel P, Mbofana F, Cousens S. The polychoric dual-componentm wealth index as an alternative to the DHS index: Addressing the urban bias. J Glob Health. 2021;11:1–19.
23. Rutstein SO. Steps to construct new DHS Wealth Index. Wealth Index Construction [Internet]. 2004;6. Available from: https://dhsprogram.com/programming/wealth index/Steps_to_constructing_the_new_DHS_Wealth_Index.pdf
24. Multilevel Statistical Models - Harvey Goldstein - Google Livres [Internet]. [cited 2023 Dec 8]. Available from: https://books.google.ml/books?hl=fr&lr=&id=mdwt7ibSGUYC&oi=fnd&pg=PP14&dq=multilevel+statistical+models+goldstein+pdf&ots=fAKoE1NS0Y&sig=CPxODEPxWes9mOmRnJMqXix-RRg&redir_esc=y#v=onepage&q=multilevel%20statistical%20models%20goldstein%20pdf&f=false
25. Hasinur Rahaman Khan Md, Shaw JEH. Multilevel Logistic Regression Analysis Applied to Binary Contraceptive Prevalence Data. Journal of Data Science. 2021;
26. Merlo J, Chaix B, Yang M, Lynch J, Råstam L. A brief conceptual tutorial of multilevel analysis in social epidemiology: Linking the statistical concept of clustering to the idea of contextual phenomenon. Vol. 59, Journal of Epidemiology and Community Health. 2005.
27. Ridde V, Antwi AA, Boidin B, Chemouni B, Hane F, Touré L. Time to abandon amateurism and volunteerism: addressing tensions between the Alma-Ata principle of community participation and the effectiveness of community-based health insurance in Africa. BMJ Glob Health [Internet]. 2018;3(Suppl 3). Available from: https://gh.bmj.com/content/3/Suppl_3/e001056
28. Chankova S, Sulzbach S, Diop F. Impact of mutual health organizations: Evidence from West Africa. Health Policy Plan. 2008;23(4).
29. Onwujekwe O, Dike N, Uzochukwu B, Ezeoke O. Informal payments for healthcare : Differences in expenditures from consumers and providers perspectives for treatment of malaria in. Health Policy (New York) [Internet]. 2010;96(1):72–9. Available from: http://dx.doi.org/10.1016/j.healthpol.2009.12.014
30. Smith K V., Sulzbach S. Community-based health insurance and access to maternal health services: Evidence from three West African countries. Soc Sci Med. 2008 Jun 1;66(12):2460–73.
31. Eze P, Ilechukwu S, Lawani LO. Impact of community-based health insurance in low- and middle-income countries: A systematic review and meta-analysis. PLoS One [Internet]. 2023

Jun 1 [cited 2023 Oct 28];18(6):e0287600. Available from: https://journals.plos.org/plosone/article?id=10.1371/journal.pone.0287600
32. Ridde V, Gaye I, Ventelou B, Paul E, Faye A. Mandatory membership of community-based mutual health insurance in Senegal: A national survey. PLOS Global Public Health [Internet]. 2023 Sep 28 [cited 2023 Dec 8];3(9):e0001859. Available from: /pmc/articles/PMC10538694/
33. Seppey M, Touré L, Valéry R. Defining an action-research's content to improve a policy supporting indigents' health in Mali: a concept mapping. J Glob Health Rep. 2021 Apr 19;5(2021).
34. Touré L, Ridde V. The emergence of the national medical assistance scheme for the poorest in Mali. 2020;
35. Ly MS, Faye A, Ba MF. Impact of community-based health insurance on healthcare utilisation and out-of-pocket expenditures for the poor in Senegal. BMJ Open [Internet]. 2022;12(12). Available from: https://bmjopen.bmj.com/content/12/12/e063035
36. Bousmah M al Q, Diakhaté P, à Dieu Toulao G, Hesran JY Le, Lalou R. Effects of a free health insurance programme for the poor on health service utilisation and financial protection in Senegal. BMJ Glob Health [Internet]. 2022;7(Suppl 9). Available from: https://gh.bmj.com/content/7/Suppl_9/e009977
37. Shewamene Z, Tiruneh G, Abraha A, Reshad A, Terefe MM, Shimels T, et al. Barriers to uptake of community-based health insurance in sub-Saharan Africa: a systematic review. Health Policy Plan. 2021;36(10):1705–14.
38. Bevans R. Akaike Information Criterion | When & How to Use It (Example). Scribbr [Internet]. 2020;1–8. Available from: https://www.scribbr.com/statistics/akaike-information-criterion/
39. Amini P, Mohammadi M, Omani-Samani R, Almasi-Hashiani A, Maroufizadeh S. Factors associated with cesarean section in tehran, Iran using multilevel logistic regression model. Osong Public Health Res Perspect. 2018;9(2):86–92.

Supporting information

Appendix S1: Statistical tests for choosing the appropriate model

The BIC and AIC criteria are mathematical methods for assessing the suitability of a model to the data from which they were generated (38). The model with smallest values of the BIC or AIC tests is the one that adjusts the data better. The LL test is a measure of the suitability of a model; the higher the value, the better the model. The ICC expresses the proportion of total variance at the common or village level (39). The greater the value of the ICC, the more the characteristics of the groups (municipalities, villages) are different, and therefore, the model with mixed effects is appropriate. The results of AIC, BIC and LL tests do not discriminate between different models.

S1 Table : Statistical tests of model comparisons

Click here to access/download
Supporting Information
declaration-of-competing-interests.docx

Click here to access/download
Supporting Information
Inclusivity-in-global-research-questionnaire.docx

Click here to access/download
Supporting Information
S1 Table - Statistical tests of model comparisons.docx
